# Colon Rectal Liver Metastases: The Role of the Liver Transplantation in the Era of the Transplant Oncology and Precision Medicine

**DOI:** 10.3389/fsurg.2021.693387

**Published:** 2021-07-20

**Authors:** Michele Finotti, Alessandro Vitale, Enrico Gringeri, Francesco Enrico D'Amico, Riccardo Boetto, Alessandra Bertacco, Sara Lonardi, Francesca Bergamo, Paolo Feltracco, Umberto Cillo

**Affiliations:** ^1^University of Padova, Transplantation and Hepatobiliary Surgery, Padua, Italy; ^2^Unit of Oncology 1, Department of Oncology, Veneto Institute of Oncology IOV - Istituto di Ricovero e Cura a Carattere Scientifico, Padua, Italy; ^3^Anesthesia and Intensive Care Unit, Azienda Ospedaliera di Padova, Padua, Italy

**Keywords:** liver resection, transplant benefit, prioritization, immunosopressive therapy, recurrence

## Abstract

The development of liver metastases in colon rectal cancer has a strong impact on the overall survival (OS) of the patient, with a 5-year survival rate of 5% with palliative treatment. Surgical resection combined with pharmacological treatment can achieve a 5-year OS rate of 31–58%. However, in only 20% of patients with colon rectal liver metastases (CRLMs), liver resection is feasible. In highly selected patients, recent trials and studies proved that liver transplantation (LT) for non-resectable CRLM is a surgical option with an excellent long-term OS. The paper aims to review the indications and outcome of LT for CRLMs, with a special focus on immunosuppressive therapy and the management of local and extrahepatic recurrence after LT.

## Introduction

Colorectal cancer (CRC) is the third most common tumor worldwide. In 20–30% of cases, the liver is affected by metastases, strongly undermining the survival of patients. If compared to other organs involvement, liver metastases are associated with a higher decrease in the overall survival (OS): The median OS is about 8–10 months, with a 5-year survival rate of 5% with palliative treatment (1).

Pharmacological therapy alone is rarely effective in the presence of ColoRectal Liver Metastases (CRLMs). The current treatment of choice for CRLMs is represented by surgical resection, combined with systemic chemotherapy, achieving a 5-year OS of 31–58% (2, 3).

Numerous surgical and radiological options are available: liver resection with tissue-sparing techniques, multistage hepatectomies, ablative therapies such as microwave or radio-frequency ablation, irreversible electroporation, regional hepatic intra-arterial chemotherapy, stereotactic radiotherapy, and radio-embolization.

Control of the local disease with an aggressive surgical approach to the CRLMs is supported by the literature: Liver partition and portal vein ligation for staged hepatectomy (ALPPS), repeated resection, or two-stage hepatectomies are especially indicated for patients with insufficient future liver remnants (4–7).

Despite the recent evolution in hepatobiliary surgery techniques, however, in almost 80% of patients with CRLMs, liver resection is still not feasible, especially due to the localization and number of nodules (3).

The definition of non-resectable liver metastases is still not well-established and under evolution.

Recent guidelines define resectable as a tumor that can be resected completely with adequate margin (R0), leaving a sufficient liver remnant with adequate vascular inflow and outflow as well as biliary drainage. In particular, localization, dimension of the metastases, the presence of extrahepatic disease, and vascular invasion (possibility of reconstruction of vena cava and/or portal vein) should no longer restrict the indication to liver resection (8).

However, the postoperative and oncological outcomes are strictly correlated with an appropriate patient selection. Biological factors, in particular tumor biology, liver tumor burden, and number of lesions, are critical factors that heavily affect the survival of a patient.

Appropriate scores can predict the OS and the risk of recurrence, helping the selection of patients, such as Fong, Nordlinger, Nagashima, and Konopke scores (9–12). High-risk patients can benefit most from neoadjuvant chemotherapy before liver resection.

In this setting and in highly selected patients, recent trials and studies proved that liver transplantation (LT) for non-resectable CRLM is a surgical option with an excellent long-term OS, offering a R0 procedure by replacing the liver (13).

The role of LT in the context of liver tumors has been recently reconsidered and is in constant evolution. According to the concept of Transplant Oncology, the long-term outcome after LT for malignant liver tumors should be similar to those of LT for the non-malignant indication (14–17).

The first series of LT for CRLM have been proposed in the 1980s and the 1990s, with poor outcomes. At the time, the peri- and postoperative mortality after LT reached 30%, the liver recurrence was described in 40% of the patients within 1 year and the 5-year OS ranged from 0 to 18%. In the 1990s, Muhlbacher et al. reported the largest series of CRLM treated with LT, with a 5-year survival rate of only 12% (18).

Due to these results, LT for CRLM was considered an unacceptable waste of resources, especially in an era of shortages of donor grafts.

Since the 1980s and 1990s, however, surgical and especially medical management dramatically changed. The improvement of pharmacological therapies for CRLM promoted the use of oxaliplatin and irinotecan in combination with monoclonal antibodies (antivascular endothelial growth factor receptor or antiepidermal growth factor receptor), reaching excellent local disease control (19–22).

A better understanding of the predictive factors associated with local recurrences and OS helped to better select patients who were eligible for LT with a good outcome. In the last decades, more refined intra- and postoperative management following LT achieved a significant reduction in the mortality and morbidity related to the surgical procedure (9, 23–25).

Furthermore, the use of immunosuppressive agents with antineoplastic abilities (mTOR—mammalian target of rapamycin inhibitors) helped to improve the patient outcome (26–28).

In our paper, we intend to review the most recent available data, indications, and outcomes of LT for CRLMs, with a special focus on immunosuppressive therapy and the management of local and extrahepatic recurrence after LT.

## The Secondary Cancer I Study and SECA II: From the Proof of Concept to the Clinical Trial

In 2013, the SECA I was the first prospective trial that reported 21 patients with liver limited disease (29). Non-resectable CRLMs were treated with LT after at least 6 weeks of neoadjuvant chemotherapy. In SECA I, the inclusion criteria was relatively broad (see Table 1). The study reported a Kaplan–Meier estimate of 1-, 3-, and 5-year OS of 95, 68, and 60%, respectively, with a median follow-up of 27 months (range: 8–60 months). Compared with mere chemotherapy (Nordic VII trial), the improvement in the OS with LT was dramatic: The 5-year OS rate was 60% in patients undergoing LT, and it was 9% in patients treated only with chemotherapy (30).

**Table 1:** Comparison of the published result on LT for CRLMs.

**Variables**		**SECA I NCT01311453**	**SECA II study NCT01479608**	**SECA II study D arm NCT01479608**	**Compagnons Hépato-Biliaires**
Type of study		Prospective pilot study	Prospective study	Prospective study	Retrospective cohort study
Country		Norway	Norway	Norway	France
Start		10/2006	5/2012	5/2012	10/1995
State		Finished	Recruiting	Recruiting	Finished
Definition of non-resectability		Based on the location of metastases and the volume of the future liver remnant	–	–	–
Number of participants		21	15	10	12
Location of the primary tumor		Colon (52%) Rectum (48%)	Colon (73.3%) Rectum (26.6%)	Colon (90%) Rectum (10%)	Colon (91.6%) Rectum (8.3%)
Stage of	T1	4.7%	6.6%	0%	8.3%
primary tumor	T2	9.5%	13.3%	20%	8.3%
	T3	76.1%	73.3%	70%	66.6%
	T4	–	6.6%	10%	16.6%
	N0	33.3%	53.3%	20%	41.6%
	N1	33.3%	40%	0%	41.6%
	N2	33.3%	6.6%	80%	16.6%
KRAS		–	7.1%	30%	–
Cancer treatments before LT	Two or more lines	57%	53.3%	100%	75%
	Principal CT	Irinotecan and oxaliplatin	5-FU and irinotecan	5-FU and irinotecan	Irinotecan and oxaliplatin
	Previous radiation	14.2%	No	10%	No
	Previous liver resection	19%	26.6%	20%	83.3%
	Previous local treatment	9.5%	13.3%	20%	8.3%
Time of liver	Metachronous	19%	6.6%	0%	25%
metastases	Synchronous	81%	93.3%	100%	75%
Time from primary surgery to LT		36 months (16–59)	22.6 months (2.3–111.3)	16.5 months (4–173)	41 months (12–97)
Type of Graft		Whole liver from deceased donor	Whole liver from deceased donor	Whole liver from extended criteria deceased donor	Whole liver from deceased domino donor
				Split liver from extended criteria deceased donor	Living donor liver transplantation
Inclusion criteria		- Completed radical excision of the primary tumor - ECOG score 0 or 1 - Minimum 6 weeks of CT. - Absence of extrahepatic disease was assessed by chest, abdominal, and pelvic computed tomographic (CT) scans, whole-body positron emission tomographic/CT scan, and bone scan.	- Standard surgical resection procedure of primary tumor with adequate resection margins, including circumferential resection margins (CRM) of at least 2mm for rectal cancer - ECOG 0 or 1 - At least 1-year time span from CRC diagnosis and date of being listed on the transplantation list. - No extrahepatic metastatic disease or local recurrence according to PET/CT scan, CT, or MR (thorax/abdomen/pelvis) scan within 4 weeks before the faculty meeting - Histologically verified adenocarcinoma in colon or rectum - No signs of local recurrence judged by colonoscopy/CT colography within 12 months before the faculty meeting - Satisfactory blood tests^*^ - Relapse of liver metastases after second liver resection or liver metastases not eligible for curative liver resection - Received first-line treatment - Before the start of CT, no lesion should be >10 cm, if >30 lesions all should be <5 cm, and the patients should have at least 30% response by RECIST criteria. - At least 10% response (RECIST criteria) on CT. - If <10% response on CT may be included if they obtain at least 20% response after TACE (DEB-IRI) or by 90Y-spheres.	The same as SECA-II, but ALSO include patients: - Who had resectable pulmonary metastases or undergone resection of pulmonary metastases - Received CT before inclusion, but there was no prerequisite regarding the response to CT at time of being listed for LT - No time from primary diagnosis to LT	- Response and no progression under CT - No signs of extrahepatic metastatic disease or local recurrence - Relapse of liver metastases after second liver resection or liver metastases not eligible for curative liver resection - Salvage procedures: massive bleeding (and packing) following liver resection, failed ALPPS procedure and for post-resection liver failure
Main exclusion criteria		- Weight loss of more than 10% - Standard contraindications for liver transplantation and/or other malignancies	- Weight loss >10% the last 6 months - BMI >30 - Other malignancies - Known hypersensitivity to rapamycin - Prior extrahepatic metastatic disease or local relapse - Not standard preoperative, peri-operative, or postoperative treatment for the primary CRC - Palliative resection of primary CRC tumor - Women who are pregnant or breast feeding - Any reason why, in the opinion of the investigator, the patient should not participate	As SECA-II	- Standard contraindications for liver transplantation and/or other malignancies
Risk stratification		The Fong Clinical Risk Score (FCRS)	FCRS and the Oslo score	FCRS and the Oslo score	–
Immunosuppressive protocol		Sirolimus, mycophenolate mofetil, corticosteroids, and induction with basiliximab. Sirolimus from the first POD, level of 5 to 10 ng/mL during the first 4 weeks and 10 to 20 ng/mL thereafter	Induction with basiliximab, tacrolimus the first 4–6 weeks, and then conversion to sirolimus.	Induction with basiliximab, corticosteroids, mycophenolate, and tacrolimus the first 4–6 weeks, then conversion from tacrolimus to sirolimus, level of 5–10 ng/mL during the first 4 weeks and 10–20 ng/mL thereafter	mTOR inhibitors in 66.6% of patients
Complications	Grade I	5%	–	20%	–
	Grade II	29%	–	20%	25%
	Grade IIIa	10%	13.3%	–	–
	Grade IIIb	24%	20%	30%	8.3%
	Grade IVa	4%	13.3%	20%	16.6%
	Grade IVb	–	–	10%	–
	Grade V	–	–	–	8.3%
Results	Median FU	27 months (8–60 months)	36 months (5–60)	23 months	26 months
	OS 1 y	95%	100%	70%	83%
	3 y	68%	83%	40%	62%
	5 y	60%	83%	–	50%
	DFS 1 y	35%	53%	30%	56%
	3 y	–	44%	20%	38%
	5 y	–	35%	–	38%
	Time to recurrence	8 months (2–24 months)	8.8 months (2.7–24.3 months)	–	–
	Site of recurrence	>pulmonary	>pulmonary	>pulmonary	>pulmonary
Post-LT treatments	Ablation, resection, radiation	Yes	Yes	Yes	Yes
	Adjuvant CT	No	No	No	Yes

* *Hb > 10 g/dL, neutrophils > 1.0 (after any G-CSF), TRC > 75, bilirubin < 2 x upper normal level, AST/ALT < 5 x upper normal level, creatinine < 1.25 x upper normal level. Albumin above lower normal level*.

*BMI, Body Mass Index; CRM, Circumferential Resection Margins; CT, Chemotherapy; CRLMs, Colo-Rectal Liver Metastases; DSF, Disease-Free Survival; ECOG, Eastern Cooperative Oncology Group; FCRS, Fong Clinical Risk Score; FU, fluorouracil; LT, liver transplant; OS, Overall Survival; POD, Postoperative Day*.

Tumor < 5.5 cm, CEA < 80 ng/L, major than 2 years between resection of primary tumor and LT, and evidence of tumor partial response or stable disease on chemotherapy have been identified as important factors leading to a better OS after LT (31).

These variables were included in the Oslo score, an important tool that helps in the stratification of the patients undergoing LT for CRLMs and identifies patients with favorable tumor biology. Oslo score numbered the risk factors from 0 to 4 stratifying the patients into three subgroups (0–1, 2–3, and 4). To note, variables included in Olso score were similar to Fong's Clinical Risk Score (FCRS) for recurrence after liver resection for CRLMs (see Table 2). Patients with an Oslo risk score of 4 had a 5-year OS of 0% (31).

**Table 2:** Oslo and Fong scores: clinical risk prognostic scores of LT for CRLMs.

**Oslo score (0–4)**	**Fong clinical risk score (FCRS) (0–5)**
Tumor diameter > 5.5 cm	Largest tumor > 5 cm
CEA > 80 ug/L	CEA > 200 ug/L
Less than 2-year interval between primary resection and LT	Synchronous Disease (primary to liver recurrence <12 months)
Progressive disease at time of LT	Node-positive primary
	More than 1 liver metastases

However, as recently acknowledged by Smedman et al., the Oslo score reveals important limitations. It does not take into account some clinicopathological features of the disease that are dramatically relevant for its prognoses, such as BRAF mutation status, histological differentiation, location of the primary tumor (right-sided, left-sided, or rectal), and node status (32).

In the SECA II, with more strictly selection criteria than SECA I (see Table 1), the authors obtained a Kaplan–Meier OS at 1, 3, and 5 years of 100%, 83, and 83%, respectively, with a median follow-up of 36 months (range 5–60 months).

The CRLMs were confirmed by CT, MRI, and positron emission tomography (18) (F-FDG PET/CT scan), with at least 10% response to CT and with a time interval between diagnoses of LT of more than 1 year (33). CT scan is more often applied compared to MRI, since thoracic staging is better evaluated with CT scan, while PET/CT scan is mandatory, considering also its prognostic value (34).

## Colon Rectal Liver Metastases as a New Indication to LT: the Challenging of the Prioritization

The SEcondary CAncer I study II and I were conducted under a unique geographical condition: The Norwegian system allows low waitlist mortality (<3%) and short waiting times. Most of the patients with high MELD get a transplant within 3 months, thanks to the good liver donation rate and the different epidemiology of liver diseases compared to the rest of the Western World (35). In the rest of the world, the indication of LT for CRLM as a viable treatment option, on the contrary, must deal with organ shortage. The major concern, therefore, was that adding a new indication for LT could affect negatively the waiting lists, already burdened by high mortality for the long waiting time. However, using strict inclusion criteria to select properly patients, the impact on the waiting list would be not unacceptable. In the United States, it was estimated that LT for CRLMs would represent ~3% of the transplants performed in the United States per year (36).

Furthermore, the expanding use of machine perfusion and the recent improvement in marginal donor management may limit liver graft shortage. An ongoing clinical trial is evaluating the utilization of extended criteria liver grafts from donors (ECD) not utilized for approved LT indications (SOULMATE, NCT04161092). Smedman et al. confirmed that, in the SECA-II D arm, the use of ECD grafts appears to be safe (32).

In this setting, two important studies proposed alternative options to increase the donor pool. The RAPID concept (37) and the use of living donor (Toronto Protocol) have been recently proposed and are currently under investigation (38).

In the RAPID concept, a left lateral split liver graft from a deceased or living donor is implanted in the recipient affected by non-resectable CRLMs after left hepatectomy through APOLT technique (auxiliary partial orthotopic LT) (39). The right portal vein is ligated to induce hypertrophy with an adequate portal and arterial flow/pressure management, and a delayed hepatectomy is performed (15, 37, 40). Ongoing clinical trials are based precisely on this technique (NCT02215889 and NCT04865471).

Inspired by the same concept, Konigsrainer et al. proposed to use the left lateral segment from living donor [LIVER-T(W)O-HEAL] (41). Living donation allows expanding the LT indication without affecting the deceased donor pool. Furthermore, the use of only later lobes reduces the risks for the donors (42).

Recently, Ravaioli et al. proposed an alternative to the RAPID technique. Their procedure consists of a heterotopic LT of segments 2–3 in the splenic fossa after splenectomy with delayed hepatectomy after regeneration of the transplanted graft. The supposed advantage of this procedure compared to the RAPID technique is no native liver manipulation and possible application in the case of previous liver resections (43, 44).

These new procedures mixed the latest development in hepatobiliary and LT surgery, using a combination of living donor liver transplantation, APOLT (39), and associating liver partition and portal vein ligation for staged hepatectomy (ALPPS) techniques (45, 46).

A comparison of the ongoing clinical trials on LT for CRLMs is summarized in Table 3.

**Table 3:** Ongoing clinical trials on LT for CRLMs.

	**Toronto NCT 02864485**	**TRANSMET NCT02597348**	**RAPID NCT02215889**	**LIVER-T(W)O-HEAL NCT03488953**	**SOULMATE NCT04161092**	**SECA III NCT03494946**	**COLT NCT03803436**	**RAPID-PADOVA NCT04865471**	**MELODIC NCT04870879**
Type of study	Prospective cohort	Prospective, multicenter randomized parallel	Prospective	Prospective bi-institutional, one-arm trial.	Randomized controlled, open-label, multicenter study	Randomized study standard	Multicenter, non-randomized	Prospective, multicenter, non-randomized	Prospective multicenter, non-randomized
Design	LDLT + CT vs. CT	CT + LT vs. CT	Liver resection and partial section S2/3 transplanted with two-stage hepatectomy	LDLT with two-stage hepatectomy	LT with ECD + CT vs. CT	LT vs. CT, TACE, SIRT or other available treatment options	LT + CT vs. CT	Liver resection and partial section S2/3 transplanted	LT + CT vs. CT
Country	Canada	France	Norway	Germany	Swedish	Norway	Italy	Italy	Italy
Start	8/2016	9/2015	6/2014	5/2018	2/2020	12/2016	1/2019	To be determined	To be determined
State	Recruiting	Recruiting	Recruiting	Recruiting	Not yet recruiting	Recruiting	Recruiting	Not yet recruiting	Not yet recruiting
Definition of non resectability	Bilateral and non-resectable CRLMs	Independent Steering Committee including hepatobiliary surgeons, oncologists, radiologists, and hepatologists	Liver metastases, not amenable to liver resection	Unresectability is evaluated by experienced, independent hepatobiliary surgeons	Patients with non-resectable, non-ablatable liver metastases	Liver metastases, not amenable to liver resection	Liver metastases not eligible for curative liver resection	Liver metastases, not amenable to liver resection	Liver metastases, not amenable to liver resection
Type of graft and donor	Living donor liver transplantation	Whole liver from deceased donor.	Living donor segment 2/3 Stage 1: S2–S3 removed and liver donor implanted. Stage 2: After growth of donor segments, the remaining liver segments of the recipient were removed	Living donor segment 2/3 with two-stage hepatectomy	Liver grafts from extended criteria donors not utilized for approved indications	Whole liver from deceased donor	LT from cadaveric donors	Living donors or cadaveric donors	LT from cadaveric donors
Main inclusion criteria	- ECOG 0-1 - Proven colorectal liver metastases - Primary tumor stage ≤ T4a - Time from primary to LT ≥6 months - No major vascular invasion - Metastases isolated to liver - Previous CT for ≥3 months with stability or regression of CRLMs - CEA values are stable or decreasing - BRAF wild-type	- ECOG 0 or 1 - ≥ 18 and ≤ 65 years - Histologically proved adenocarcinoma in colon or rectum - BRAF wild-type - High-standard oncological surgical resection of the primary^*^ - No local recurrence on colonoscopy - ≥ 3 months of tumor control during the last CT line: stable or partial response on RECIST criteria - ≤ 2 lines of CT for metastatic disease - CEA <80 microg/L or a decrease ≥ 50% - No extrahepatic tumor	- ECOG 0 or 1 - Histologically proved adenocarcinoma in the colon or rectum - No extrahepatic or local recurrence (except 1–3 resectable lung lesions all <15 mm) - At least 8 weeks of CT	- Stable disease after 8 weeks of CT - Primary tumor < pT3, N1 - No extrahepatic tumor burden (except resectable lung metastases)	- ECOG 0 or 1 - ≥ 18 years - Histologically proved adenocarcinoma in the colon or rectum - Primary tumor removed with an R0 resection - No extrahepatic or local recurrence - At least 2 months of CT with no signs of progression - >1 year from initial diagnosis - BRAF wild-type	- ECOG 0 or 1 - Histologically proved adenocarcinoma in the colon or rectum - No extrahepatic or local recurrence (except resectable lung lesions all <15 mm). - All patients should have progressive disease according to RECIST criteria, or intolerance to 1. line CT. - Patients must be randomized before evaluation 8–12 weeks after starting the second-line chemotherapy	- ECOG 0 - Histologically confirmed non-mucinous colon adenocarcinoma - pT1-3, pN0 or pN1 (metastases in <4 regional lymph nodes), confirmed R0 resection. - RAS and BRAF wild-type - Objective response according to RECIST 1.1 to first-line treatment, with sustained response for at least 4 months, OR disease control (CR + PR + SD) during the second-line treatment for at least 4 months. - A maximum of two prior CT treatment lines. - CEA <50 ng/ml	- ECOG 0 or 1 - ≥ 18 and <70 years - BRAF wild-type - High standard oncological surgical resection of the primary^*^ - Histologically proved adenocarcinoma in the colon or rectum - Time from primary surgery to WL ≥6 months - At least 2 months of stable disease or partial response according to RECIST 1.1 - At least 1 CT line treatment (3 months) - CEA stability or reduction - No extrahepatic disease, expect for lung metastases (up to three resectable nodules or liable to radiotherapy with no progression after 3 months) - Resected hilar hepatic adenopathy with no progression after 3 months	- ECOG 0 or 1 - ≥ 18 and <70 years - High-standard oncological surgical resection of the primary^*^ - BRAF wild-type - At least 1 CT line treatment (3 months) - No extrahepatic or local recurrence - No hepatic lesions > 10 cm before CT treatment - Tumor response according to RECIST 1.1 on two stadiation with no CEA elevation - If tumor response after CT <10%, only in 20% tumor reduction after TACE (DEB-IRI) o 90Y-spheres - Time from primary surgery to WL ≥10 months - CEA <100 ng/ml
Main exclusion criteria	- General contraindication to LT - Prior lung resection - Progression of CRLMs at any timepoint prior to LT - History of HIV or chronic HBV/HCV.	- General contraindication to LT - Patients not having received standard treatment for the primary CRC - Extrahepatic metastatic disease or local relapse	- General contraindication to LT - Weight loss >10% the last 6 months - BMI > 30 - Palliative resection of primary CRC tumor.	- General contraindication to LT - Macroscopic vascular tumor infiltration - Tumor progression during CT	- General contraindication to LT - Extrahepatic disease - Weight loss >10% the last 6 months - Liver metastases larger than 10 cm. - Pathological lymphatic nodes in the abdomen. - Microsatellite instability—previous transplantation	- General contraindication to LT - Weight loss >10% the last 6 months - BMI > 30 - Resection of local relapse or non-hepatic metastasis within 2 years or resection of pulmonary/liver hilus lymph node metastases <1 year - Liver lesion>10cm - 3 negative prognostic factors (CEA>80, less than 2 years from diagnosis, diameter of largest liver lesion > 5.5 cm).	- General contraindication to LT - Hereditary CRC syndromes - Extrahepatic metastatic disease or primary tumor local relapse. - Active intravenous or alcohol abusers - HIV infection	- General contraindication to LT - Patients not having received standard treatment for the primary CRC - BMI > 30 - Weight loss >10% the last 6 months	- General contraindication to LT - Patients not having received standard treatment for the primary CRC - BMI > 30 - Weight loss >10% the last 6 months

* *defined by : Safe margin of resection, curative resection of primary tumor according to oncological principles and TNM adequate staging*.

*BMI, Body Mass Index; CT, Chemotherapy; ECD, Extended Criteria Donor; ECOG, Eastern Cooperative Oncology Group; LDLT, Living Donor Liver Transplantation; LT, Liver Transplant*.

## Management of the Immunosuppressive Therapy and Recurrences

Metastatic CRC is considered a systemic disease, and the risk of recurrence after surgical therapy is high. Twenty percent of patients affected by CRLMs are liable to liver resections (47) but after resection, up to 60–70% of them will develop recurrence within 3 years (48–50).

Starting from this premise, recurrence after LT is expected. In addition, immunosuppressive therapy after LT has been thought to predispose to malignancy and recurrence after LT. Immunosuppression, in particular, mTOR (a mammalian target of rapamycin) inhibitors, has been correlated with incisional hernia, HAT, rejections, and risk of cancer development.

In the SECA-I and SECA-II, mTOR inhibitor was the main immunosuppressive agent used. HAT, potentially related to mTOR inhibitors use, was observed only in one patient. The increase of postoperative anticoagulation restricted the arterial complications to the technical aspect, leading, however, to increasing episodes of hemorrhage and hematomas (31). The role of immunosuppression in the development of metastases has been evaluated, pointing out a comparable growth rate of the metastases in immunosuppressed and immunocompetent patients (51). However, more data will be necessary to understand the role of immunosuppression and the relationship with recurrence after LT.

To note, the pattern of recurrence and their natural history after LT is different compared to that of recurrence after liver resection for CRLM (33).

After hepatectomies for CRLM, most of the recurrence appeared within the remnant liver, with only 26% of patients developing isolated lung metastases (6). On the other side, after LT, the recurrence is mostly pulmonary, isolated, and liable to curative surgery (13, 52, 53). These results suggest that total hepatectomy and LT may have a favorable recurrence profile compared to recurrence after liver resection (51).

In the SECA I, the median time of recurrence was 8 months (range, 2–24 months), reflected by the low disease-free survival (DFS). However, most of the patients benefit from surgical, radio-frequency, or radiation treatment of the local recurrence with curative intent. Pulmonary metastases are the most frequent site of progression, but are usually solitary with slow development (31). In the SECA II, with more strict inclusion criteria, four of the 15 patients were observed for more than 30 months without a relapse, and pulmonary recurrences are the only or primary location in 75% of patients. The same study confirmed the slow growth rate of the recurrence, especially for the pulmonary metastases, with a median time of 21 months from detection to resection.

Based on these preliminary data, the recurrence after LT for CRLMs seems to have different behavior and impact on the patient outcome compared, for example, to recurrence in patients affected by HCC. Based on the SECA-I study, Dueland et al. showed that in a low-risk patient who underwent LT for CRLM with subsequent recurrence, the OS was better than in patients affected by recurrence after LT for HCC (54).

DFS in the LT for CRLMs is not a good tool to predict the patient outcome and the efficacy of LT in CRLMs, because recurrence alone is not correlated with low OS (33).

Recently, Compagnons Hepato-Biliaires demonstrated that, with carefully selected patients, a long-term DFS can be achieved. The authors reported that six of 12 recipients alive free of recurrence at 7–108 months after LT for CRLMs. In these patients, multiple courses of CT and liver resection were performed before LT. Conversely, the other six of 12 patients that undergone LT for “compassionate” indications (LT after massive bleeding, post-resection liver failure) showed recurrence within 18 months with a significantly worse DFS. In the whole cohort, the OS was 83 ± 11%, 62 ± 15%, and 50 ± 16% at 1, 3, and 5 years, after a median follow-up of 26 (0–108) months. As for SECA study, the predictors of recurrence were the last pre-transplant CEA level (≥80 vs. <80 μg/l) and the time between resection of primary cancer and transplantation (≥24 vs. <24 months). To note, the size of the largest metastasis at transplant (≥5.5 vs. <5.5 cm) failed to predict DFS (55).

## Conclusion

One of the major controversies is how to integrate the LT for CRLM in the context of numerous available surgical options (local liver treatments, hepatic resection, chemo-, or radio-embolization), how to choose the best one or the best combination of them, and how to establish that the CRLM isn't resectable and liable to LT. A recent multidisciplinary consensus from the COLLISION Trial Group created an algorithm with specific resectability and ablatability criteria for the treatment of CRLMs (56). S. Jegatheeswaran and A. Siriwardena in their comment on the SECA-II study pointed out that the concept of resectability is prone to a variation. In the SECA-II, 60% of patients had five or fewer metastases with five patients having three or fewer lesions, but considered unresectable due to local recurrence after previous multiple treatments (liver resection and/or ablation) (57). However, the author acknowledged the possible variable interpretation of liver resectability (57, 58). The same concern was raised by Azoulay and Lim (36).

Recently, a survey among the most expert liver surgeons from 23 countries has been published, evaluating the different surgical approaches among them. Importantly, 68% of them have hepato-pancreatic biliary surgery and LT experiences. In particular, the aforementioned surgeons examined 10 cases of patients with CRLMs, from single metastasis to diffuse bilobar lesions. A low level of agreement was observed among them. For the same case, surgeons suggested treatments ranging from the choice of minimally invasive liver resections to complex staged liver resection. The study underlines the current difficulty to choose the best option treatments and especially the lack of consensus about the definition of resectability.

This variability could be explained because of the deficiency of high evidence studies and guidelines, and the expertise and experiences of different surgeons (59), even if non-resectability is determinant only in the context of organ shortage.

The main factor that indicates the benefit of LT for CRLMs is OS, which means that patients could benefit from LT no matter non-resectability. Resectability can be achieved in bilobar and multiple CRLMs by various complex techniques, including liver augmentation (portal vein embolization, liver venous deprivation, ALPPS). However, the outcome after resection is still highly variable, and OS is greatly affected by hepatic tumor load and the number of CRLMs (60–62). For example, ALPPS has the potential to increase the rate of resectability, but the 5-year OS is reported to be 30% compared to 83% in the SECA-II and 60% in the SECA-I study (36, 63). This leads to the interesting scenario wherein future LT may become the first treatment of choice regardless of the technical resectability of the tumor in selected patients. For example, it has recently been shown that LT in non-resectable CRLMs is superior to portal vein embolization and liver resection in resectable disease in patients with high tumor load (64).

The resectability of the CRLMs is not the only variable to take into account when LT is considered as a treatment option. The correct selection of patients affected by CRLM is the cornerstone to achieve the best benefit from LT, as for LT for HCC. SECA-I, with relatively broad inclusion criteria, obtained a 60% 5-year OS; in the SECA-II, with more strict criteria, the 5-year OS increased to 83%. In a recent subanalysis of the SECA-I and SECA-II patients, Dueland et al., compared the Fong Clinical Risk Score, total PET liver uptake (metabolic tumor volume, MTV), and Oslo score in terms of OS, DFS, and survival after relapse. Considering 13 of the 19 patients (14 and five patients from SECA-I and SECA-II), a Kaplan–Meier OS of 100, 78, and 67% at 5 years was obtained in patients with Fong Clinical Risk Score 0–2, MTV-low group, and Oslo score 0–2, respectively, with a median follow-up of 85 months (65).

Narrowing the inclusion criteria leads to a better patient selection and outcome and to the detriment of patients that would be eligible to LT. As for HCC, the selection of which patient would benefit most from LT is still a matter of debate (65–67), and transplant benefit could be the guide to the correct patient selection for LT (65, 68–70). The Oslo score proposed in the SECA study has to be integrated and validated in the ongoing trials, to define the ideal candidate for LT for CRLM.

On the other hand, the expansion of the inclusion criteria leads inevitably to a worsening of the OS after LT. Recently, Smedman et al. published a series derived from the SECA-II study (arm D) with 10 patients excluded from the SECA-II criteria. In arm D, the main patients who had resectable pulmonary metastases and no response to CT were also eligible for inclusion. Most of the patients had a pT3, pN2, poorly differentiated, right-sided primary tumor, and nine of 10 patients were transplanted with ECD grafts (32).

Two patients had a BRAF mutation, and two had a progression of the disease on the last line of chemotherapy at the time of LT. In this setting, compared to the result of the SECA-I and II, the worsening of the prognosis was dramatic: The median DFS and median OS were 4 and 18 months, respectively.

To note, the Fong score of SECA arm D was similar to that of SECA-I, and patients with an Oslo score of 1–2 had significantly shorter OS than patients with a similar Oslo score in the SECA-I study.

This is further evidence that we should take into account features different from those described by Fong and Oslo scores, such as tumor location, histological differentiation, and lymph node status of the primary tumor (32).

Another point of discussion is the role and the combination of LT and chemotherapy. In the SECA-II, the adjuvant CT was not evaluated, based on the results of the EORTC 40983 study (48). The study showed no difference in OS with the addition of adjuvant CT with FOLFOX compared with surgery alone for patients with resectable CRLMs. In the SECA-II, most of the patients received extensive neoadjuvant CT especially with oxaliplatin and 5-FU regimens, which are the only chemotherapy drugs that significantly improved the OS in the CRLMs. However, in the SECA-II, the authors state that based on the EORTC 40983 study and the extensive pre-LT CT regimens, adding 5-FU or 5-FU combined with oxaliplatin would have had not to impact on the OS, increasing only the toxicity post-LT. However, as S. Jegatheeswaran and A. Siriwardena noted, the patient in the SECA-II cohort experienced a more aggressive approach than in the EORTC 40983 study, treating pulmonary metastases with resection and performing surgical regional lymphadenectomy at LT, making the two cohorts difficult to compare (57, 58). As noted by Gorgen et al., the good results in terms of OS should also be evaluated considering the role of chemotherapy. In the SECA-II, the median number and the maximum diameter of the CRLMs consistently decreased after neoadjuvant chemotherapy (from 12 to 5 lesions and from 4.5 to 2.5 cm, respectively). The downstaging was confirmed at pathological evaluation after LT (71). However, the impact and the correct timing of CT before and after LT are still under evaluation, and to date, no sufficient data are available to make some recommendations. A trial that is evaluating the role of adjuvant CT after LT for CRLMs is ongoing (Transmet, NCT 02597348).

Furthermore, the OS of patients affected by non-resectable CRLMs treated with only CT is low, but it is unknown the results of the CT in the same highly selected patients selected for LT (36).

Compagnons Hepato-Biliaires described a long-term DFS after LT for CRLMs with patients (four of 12 patients) treated with post-transplant adjuvant chemotherapy, oxaliplatin, and/or irinotecan, including CT (55).

Recently, Brandi et al. reported the good outcome and safety of three patients treated after LT for CRLMs with chemotherapy, including cytotoxic doublet or triplet (e.g., FOLFOX, FOLFOXIRI).

The concomitant immunosuppressive use did not seem to interfere with compliance to chemotherapy (72).

To conclude, the role of LT in the treatment of CRLMs is still under evaluation, with a series of clinical trials ongoing. Recently, national and international guidelines are taking into account this therapeutic option: The ILTS Transplant Oncology Consensus Conference and the Spanish Society of Liver Transplantation published their recommendations for LT for CRLMs (73, 74), and the treatment algorithm of CRLMs has been recently proposed (75) (see Table 4).

**Table 4:** Recent guidelines and recommendations on LT for CRLMs.

**ILTS transplant oncology consensus conference (74)**	**Grade of recommendation**	**Recommendations by the Spanish Society of Liver Transplantation (73)**	**Grade of recommendation**
LT can be a viable option in highly selected patients with unresectable CRLM with only liver involvement	moderate evidence and moderate recommendation	In selected patients with unresectable liver metastases of colorectal cancer, LT could be considered only in the context of well-designed clinical trials and after a close evaluation by a multidisciplinary team composed by oncologists, hepatologists, and surgeons.	2B
LT for CRLM with low Oslo score ≤2 may improve the 5-year OS rates over those achieved with the current standard of care	moderate evidence and moderate recommendation	The optimal strategy for waiting list prioritization is not established and should be tailored according to the composition and length of the waiting list in each region.	2C
Minimization of immunosuppression is recommended	low evidence and moderate recommendation	The type of donor would be at the discretion of each transplant center according to local experience and length of stay in the waiting list.	2C
Aggressive treatment of all post-LT resectable recurrences is recommended	low evidence and moderate recommendation		
There is a need for an international registry to coordinate data collection and design further studies on LT for CRLM	moderate evidence and moderate recommendation		

*CRLM, Colon Rectal Liver Metastases; LT, Liver Transplantation; OS, Overall Survival*.

## Author Contributions

MF involved in study design and drafting of the manuscript. AV and UC revised the manuscript. EG, FD'A, RB, AB, SL, FB, and PF reviewed and approved the manuscript. All authors contributed to the article and approved the submitted version.

## Conflict of Interest

The authors declare that the research was conducted in the absence of any commercial or financial relationships that could be construed as a potential conflict of interest.
